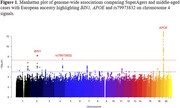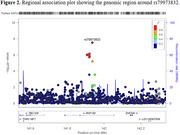# Genetic variants linked to cognitive longevity in SuperAgers

**DOI:** 10.1002/alz70855_107245

**Published:** 2025-12-24

**Authors:** Alaina Durant, Skylar Walters, Emily R. Mahoney, Shubhabrata Mukherjee, Michael L. Lee, Seo‐Eun Choi, Phoebe Scollard, Brandon Klinedinst, Emily H. Trittschuh, Jesse Mez, Lindsay A. Farrer, Katherine Gifford, Carlos Cruchaga, Jason J. Hassenstab, Adam C. Naj, Li‐San Wang, Sterling C. Johnson, Corinne D. Engelman, Walter W. Kukull, C. Dirk Keene, Andrew J. Saykin, Michael L Cuccaro, Brian W Kunkle, Margaret Pericak‐Vance, Eden R. Martin, David A. A. Bennett, Lisa L. Barnes, Julie A Schneider, William S Bush, Jonathan L Haines, Richard Mayeux, Badri N. Vardarajan, Marilyn S. S. Albert, Paul M. Thompson, Angela L. Jefferson, Paul K Crane, Logan Dumitrescu, Derek B. Archer, Timothy J. Hohman, Leslie S. Gaynor, The Alzheimer's Disease Neuroimaging Initiative (ADNI)*; Alzheimer's Disease Genetics Consortium (ADGC); The Alzheimer's Disease Sequencing Project (ADSP)

**Affiliations:** ^1^ Vanderbilt University Medical Center, Nashville, TN, USA; ^2^ Vanderbilt Memory & Alzheimer's Center, Vanderbilt University Medical Center, Nashville, TN, USA; ^3^ Department of Medicine, University of Washington, Seattle, WA, USA; ^4^ VA Puget Sound Health Care System, Seattle Division, Seattle, WA, USA; ^5^ Department of Psychiatry and Behavioral Sciences, University of Washington School of Medicine, Seattle, WA, USA; ^6^ Department of Neurology, Boston University Chobanian & Avedisian School of Medicine, Boston, MA, USA; ^7^ Biomedical Genetics, Department of Medicine, Boston University Medical School, Boston, MA, USA; ^8^ Department of Biostatistics, Boston University School of Public Health, Boston, MA, USA; ^9^ Boston University Chobanian & Avedisian School of Medicine, Boston, MA, USA; ^10^ NeuroGenomics and Informatics Center, Washington University School of Medicine, St. Louis, MO, USA; ^11^ Washington University School of Medicine, St. Louis, MO, USA; ^12^ Department of Neurology, Washington University School of Medicine in St. Louis, St. Louis, MO, USA; ^13^ Department of Biostatistics, Epidemiology, and Informatics, and the Penn Neurodegeneration Genomics Center, Department of Pathology and Laboratory Medicine, University of Pennsylvania Perelman School of Medicine, Philadelphia, PA, USA; ^14^ Department of Pathology and Laboratory Medicine, University of Pennsylvania Perelman School of Medicine, Philadelphia, PA, USA; ^15^ Alzheimer's Disease Research Center, University of Wisconsin‐Madison School of Medicine and Public Health, Madison, WI, USA; ^16^ Department of Medicine, University of Wisconsin‐Madison School of Medicine and Public Health, Madison, WI, USA; ^17^ Wisconsin Alzheimer's Disease Research Center, University of Wisconsin School of Medicine and Public Health, Madison, WI, USA; ^18^ Department of Population Health Sciences, University of Wisconsin School of Medicine and Public Health, Madison, WI, USA; ^19^ Department of Epidemiology, School of Public Health, University of Washington, Seattle, WA, USA; ^20^ Department of Laboratory Medicine and Pathology, University of Washington, Seattle, WA, USA; ^21^ Department of Medical and Molecular Genetics, Indiana University School of Medicine, Indianapolis, IN, USA; ^22^ Department of Radiology and Imaging Sciences, Indiana University School of Medicine, Indianapolis, IN, USA; ^23^ John P. Hussman Institute for Human Genomics, University of Miami Miller School of Medicine, Miami, FL, USA; ^24^ Rush Alzheimer's Disease Center, Rush University Medical Center, Chicago, IL, USA; ^25^ Cleveland Institute for Computational Biology, Department of Population and Quantitative Health Sciences, Case Western Reserve University, Cleveland, OH, USA; ^26^ The Taub Institute for Research on Alzheimer's Disease and The Aging Brain, Columbia University Medical Center and The New York Presbyterian Hospital, New York, NY, USA; ^27^ Department of Neurology, The Taub Institute for Research on Alzheimer's Disease and The Aging Brain, Columbia University Medical Center and The New York Presbyterian Hospital, New York, NY, USA; ^28^ Department of Neurology, The Johns Hopkins University School of Medicine, Baltimore, MD, USA; ^29^ Keck School of Medicine, University of Southern California, Los Angeles, CA, USA; ^30^ Department of Neurology, Vanderbilt University Medical Center, Nashville, TN, USA; ^31^ Department of General Internal Medicine, University of Washington School of Medicine, Seattle, WA, USA; ^32^ Vanderbilt Genetics Institute, Vanderbilt University Medical Center, Nashville, TN, USA; ^33^ Vanderbilt Memory & Alzheimer's Center, Vanderbilt University Medical Center, Nashville, TN, USA; ^34^ Division of Geriatric Medicine, Department of Medicine, Vanderbilt University Medical Center, Nashville, TN, USA

## Abstract

**Background:**

“SuperAgers” are oldest‐old (ages 80+) adults with memory performance resembling adults in their 50s to mid‐60s. This study investigates the genetic drivers of SuperAging using a genome wide association study (GWAS).

**Method:**

Harmonized, longitudinal memory, executive function, and language scores for participants with European ancestry were obtained from the ADSP Phenotype Harmonization Consortium. In addition to exceptional memory, SuperAgers (*N* = 1,171) must have language and executive function domain scores within normal limits and remain cognitively normal across visits if longitudinal datapoints were available. We compared SuperAgers to Alzheimer's disease (AD) cases (*N* = 5,372) and controls (*N* = 4,012) in age‐defined subgroups (middle‐aged=ages 50‐64, old=ages 65‐79, oldest‐old=age 80+). We performed a logistic regression based GWAS comparing SuperAgers and their counterparts, adjusting for age, sex, education, and the first five principal components for population substructure.

**Result:**

Comparing SuperAgers with middle‐aged cases (ages 50‐64, Figure 1), multiple variants in the confirmed AD loci APOE and *BIN1* regions were associated with genome‐wide significance (GWS; indexes *p* = 1.12×10^‐41^ and *p* = 5.48×10^‐9^, respectively). Additionally, we observed GWS association on chromosome 4 loci (rs79973832, index *p* = 2.95×10^‐8^, Figure 2), which centers on a ring finger protein gene, RNF150. This family of genes are involved in the ubiquitin‐proteasome system and regulate antiviral immune responses. While several members of the RNF gene family have been liked to AD and cognitive performance, there is currently no established association between *RNF150* and AD. Analyses comparing SuperAgers to old and oldest‐old cases (ages 65‐79 and 80+, respectively) found GWS only in the *APOE* region (indexes *p* = 1.90×" role="presentation" style="‐webkit‐user‐drag: none; ‐webkit‐tap‐highlight‐color: transparent; margin: 0px; padding: 0px; user‐select: text; display: inline‐table; font‐style: normal; font‐weight: normal; line‐height: normal; font‐size: 16px; font‐size‐adjust: none; text‐indent: 0px; text‐align: center; text‐transform: none; letter‐spacing: normal; word‐spacing: normal; overflow‐wrap: normal; white‐space: pre !important; float: none; direction: ltr; max‐width: none; max‐height: none; min‐width: 0px; min‐height: 0px; border: 0px; position: relative;" tabindex="0">×10^‐80^ and *p* = 2.53×10^‐13^, respectively). No controls had GWS associations, with the strongest associations inconsistent among age group comparisons.

**Conclusion:**

Our extreme‐phenotype GWAS comparing SuperAgers to middle‐aged cases identified both the established *APOE* and BIN1 genes and the novel loci rs79973832 for AD. Both Old Cases and Oldest‐old Cases reached GWS only in the *APOE* region. With additional harmonization efforts, larger sample sizes will allow for better comprehension of the genetic architecture of SuperAging. Future work will extend to rare variant analyses of SuperAging using Whole Genome Sequencing (WGS).